# Marine organism sulfated polysaccharides exhibiting significant antimalarial activity and inhibition of red blood cell invasion by *Plasmodium*

**DOI:** 10.1038/srep24368

**Published:** 2016-04-13

**Authors:** Joana Marques, Eduardo Vilanova, Paulo A. S. Mourão, Xavier Fernàndez-Busquets

**Affiliations:** 1 Nanomalaria Group, Institute for Bioengineering of Catalonia (IBEC), Barcelona, Spain; 2 Barcelona Institute for Global Health (ISGlobal), Barcelona Center for International Health Research (CRESIB, Hospital Clínic-Universitat de Barcelona), Barcelona, Spain; 3 Nanoscience and Nanotechnology Institute (IN2UB), University of Barcelona, Spain; 4 Hospital Universitário Clementino Fraga Filho and Instituto de Bioquímica Médica, Universidade Federal do Rio de Janeiro, Brazil

## Abstract

The antimalarial activity of heparin, against which there are no resistances known, has not been therapeutically exploited due to its potent anticoagulating activity. Here, we have explored the antiplasmodial capacity of heparin-like sulfated polysaccharides from the sea cucumbers *Ludwigothurea grisea* and *Isostichopus badionotus*, from the red alga *Botryocladia occidentalis*, and from the marine sponge *Desmapsamma anchorata*. *In vitro* experiments demonstrated for most compounds significant inhibition of *Plasmodium falciparum* growth at low-anticoagulant concentrations. This activity was found to operate through inhibition of erythrocyte invasion by *Plasmodium*, likely mediated by a coating of the parasite similar to that observed for heparin. *In vivo* four-day suppressive tests showed that several of the sulfated polysaccharides improved the survival of *Plasmodium yoelii*-infected mice. In one animal treated with *I. badionotus* fucan parasitemia was reduced from 10.4% to undetectable levels, and Western blot analysis revealed the presence of antibodies against *P. yoelii* antigens in its plasma. The retarded invasion mediated by sulfated polysaccharides, and the ensuing prolonged exposure of *Plasmodium* to the immune system, can be explored for the design of new therapeutic approaches against malaria where heparin-related polysaccharides of low anticoagulating activity could play a dual role as drugs and as potentiators of immune responses.

Among the infectious diseases, malaria ranks probably first in the perversity of its causal agent, the protist *Plasmodium spp.* This parasite distributes its life cycle^1^ between two hosts, humans and the females of certain species of mosquitoes from the genus *Anopheles*. Following a mosquito bite, in a matter of minutes sporozoites enter hepatocytes, where they will develop and replicate into thousands of merozoites that are released into the blood circulation to invade red blood cells (RBCs). Because RBCs are unable to process and present antigens, early intraerythrocytic ring stages remain invisible to the immune system until the late stages trophozoites and schizonts develop and significantly modify the parasitized RBC (pRBC) plasma membrane to meet their needs for membrane transport processes^2^. Even then, the proteins exported to the pRBC plasma membrane have a very high antigenic variation^3^ which leads to waves of parasitemia and persistent infections despite antibody-mediated immune pressure. Erythrocytes infected with mature stages of the malaria parasite bind to endothelial cells in the capillaries of tissues in a phenomenon known as sequestration, which allows *Plasmodium* to replicate while evading splenic clearance^1^. pRBCs can also adhere to non-infected RBCs giving rise to rosettes^4^, and they can form clumps through platelet-mediated binding to other pRBCs. These events, which may lead to occlusion of the microvasculature, are thought to play a major role in the fatal outcome of severe malaria. Because the blood-stage infection is responsible for all symptoms and pathologies of the disease, pRBCs have traditionally been a main chemotherapeutic target^5^. However, the fast evolution of *Plasmodium* resistance against virtually every new drug being deployed^6^, calls for urgent efforts in the research and development of new antimalarial therapeutic agents.

Negatively charged polysaccharides, such as heparin, chondroitin and dextran sulfates, fucoidan, and the nonsulfated glycosaminoglycan (GAG) hyaluronan, block cytoadhesion of pRBCs to various host receptors^7^,^8^,^9^,^10^ and disrupt *P. falciparum* rosettes^11^,^12^. Heparin and related sulfated polysaccharides possess antimalarial activity that has been described to operate through inhibition of RBC invasion by merozoites^9^,^13^,^14^,^15^,^16^. Remarkably, efforts to select for heparin-resistant parasites have proven unsuccessful^16^, which places sulfated polysaccharides as interesting candidates in the race for finding efficient long-lasting antimalarials. Proteomic analysis has revealed that heparin interacts with multiple apical surface proteins in *P. falciparum* merozoites^17^,^18^, likely blocking their association with the erythrocyte membrane after initial attachment. Naturally acquired immunity to malaria is largely directed against extracellular merozoites^19^, but currently there are no drugs targeting erythrocyte invasion by *Plasmodium*^20^, although some candidates have been proposed^21^. The potential use of heparin as drug in malaria therapy^22^,^23^,^24^,^25^,^26^ has been hindered by its high anticoagulation and bleeding properties^27^ and by the potential risk of infection since some GAGs are obtained from mammals. As an interesting alternative, non-mammalian marine organisms are a rich source of unique sulfated polysaccharides, some of them with structures resembling pRBC-binding GAGs^28^,^29^,^30^. The fucosylated chondroitin sulfate (FucCS) from the echinoderm *Ludwigothurea grisea* had been shown to have serpin-unrelated anticoagulant properties^31^,^32^, which differ from the serpin-dependent anticoagulant mechanism of mammalian heparins. Former data reporting dissociation of the anticoagulant, bleeding, and antithrombotic effects of *L. grisea* FucCS^33^, together with recent results revealing its inhibition of *P. falciparum* cytoadhesion and growth^14^, suggest that marine sulfated glycans might offer interesting alternatives to heparin for future antimalarial therapies. To trace correlations between the structure of these new sulfated polysaccharides and their inhibition of *Plasmodium* growth, we have examined several compounds containing sulfated fucose units in a well-defined repetitive sequence; namely, we have determined the antimalarial and anticoagulating activities of FucCSs and sulfated fucans from the sea cucumbers *L. grisea*^29^,^34^ and *Isostichopus badionotus*, of a sulfated galactan from the red alga *Botryocladia occidentalis*, and of a sulfated glycan from the marine sponge *Desmapsamma anchorata*.

## Results

### Characterization of sulfated polysaccharide size and integrity

**Figure 1:**
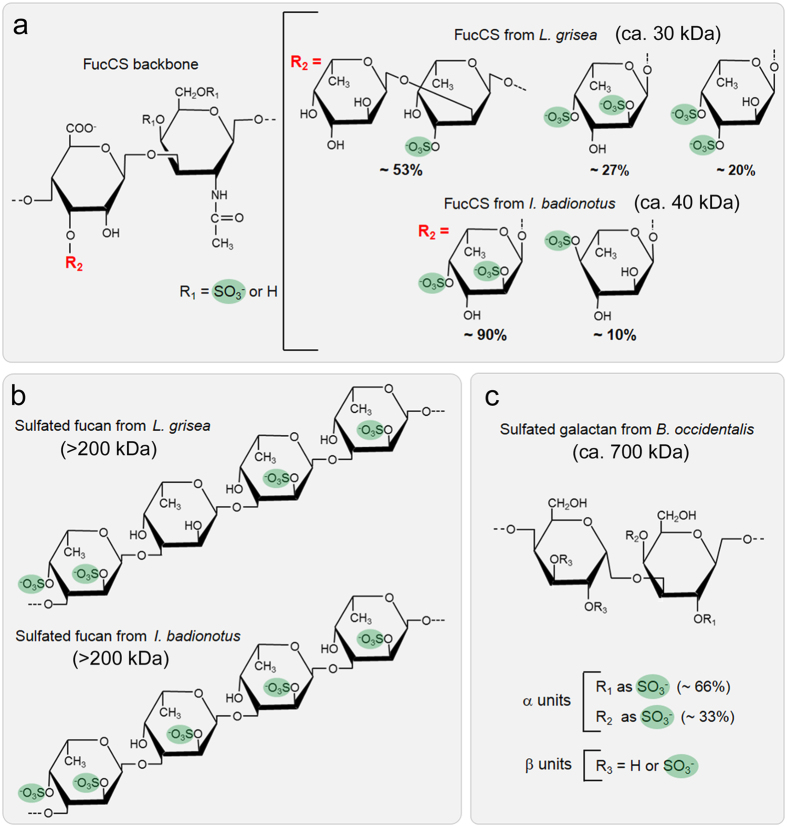
Chemical structures of the sulfated polysaccharides used in this work. **(a)** The *L. grisea* and *I. badionotus* fucosylated chondroitin sulfates share a similar backbone (left) but differ on their sulfated fucose branches (right). **(b)** *L. grisea* and *I. badionotus* sulfated fucans have similar tetrasaccharide repeating structures but differ exclusively on the sulfation of the second unit. **(c)** The sulfated galactan from the red alga *B. occidentalis* contains alternating α- and β-galactose units with distinct sulfation patterns. Sulfation sites are shadowed.

**Figure 2:**
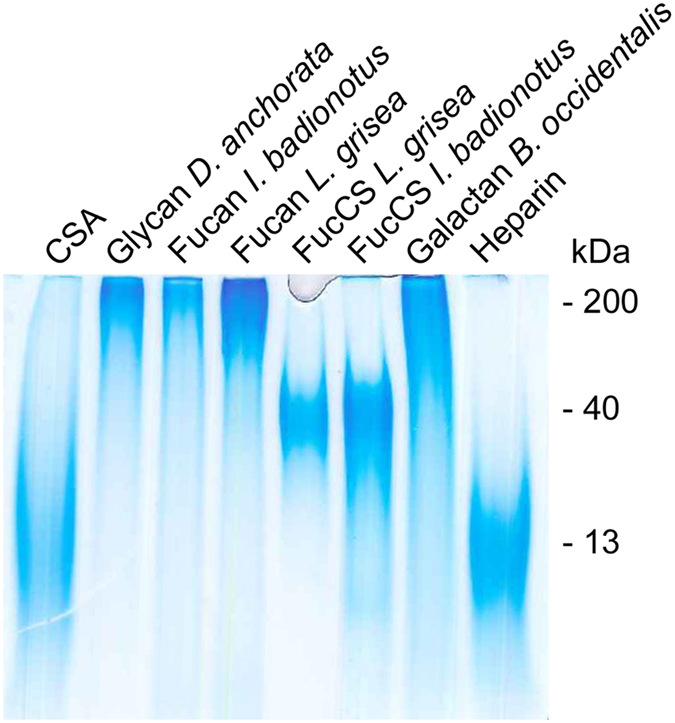
Alcian Blue-stained polyacrylamide gel electrophoresis analysis of sulfated polysaccharides. Approximate molecular masses were confirmed by size exclusion chromatography. CSA: chondroitin sulfate A.

*I. badionotus* FucCS has simple branches of sulfated α-fucose (Fig. 1a), composed of a single monosaccharide unit, either disulfated at positions 2 and 4 (~90%) or exclusively 4-sulfated (~10%)^35^. *L. grisea* FucCS has more complex branching structures, mostly composed of disaccharide units of α-fucose, non-sulfated and 3-sulfated at the nonreducing and reducing ends, respectively. This FucCS also has small amounts of branches composed of single α-fucose units, either 2,4-disulfated (~27%) or 2,3-disulfated (~20%)^35^. The linear sulfated fucans from these echinoderms (Fig. 1b) contain repetitive tetrasaccharide sequences, defined by the patterns of sulfation at positions 2 and 4^36^,^37^, which differ exclusively in the sulfation of the second residue of the tetrasaccharide: 2-sulfated in *I. badionotus* and non-sulfated in *L. grisea*. Unlike the majority of sulfated galactans from red algae, that of *B. occidentalis* (Fig. 1c) has a relatively simple structure, varying only in the sulfation pattern of its units^38^. The sponge glycan used here (*M*r ~200 kDa) has had its structure only partially elucidated; preliminary gas chromatography/mass spectroscopy analysis indicated that it is a heteropolysaccharide composed of glucose (75%), fucose (17%) and galactose (8%), with a molar ratio sulfate:total monosaccharide of ~1.5 (data not shown). Integrity of these molecules was analyzed by polyacrylamide gel electrophoresis (Fig. 2), and the result obtained was found to be consistent with the respective approximate molecular masses calculated by size exclusion chromatography after polysaccharide purification from their natural sources, indicating the absence of significant degradation. The bands observed in 6% polyacrylamide gels exhibited a certain degree of size polydispersity, as it is typical of this group of compounds.

### Antimalarial and anticoagulating activities *in vitro* of sulfated polysaccharides

**Figure 3:**
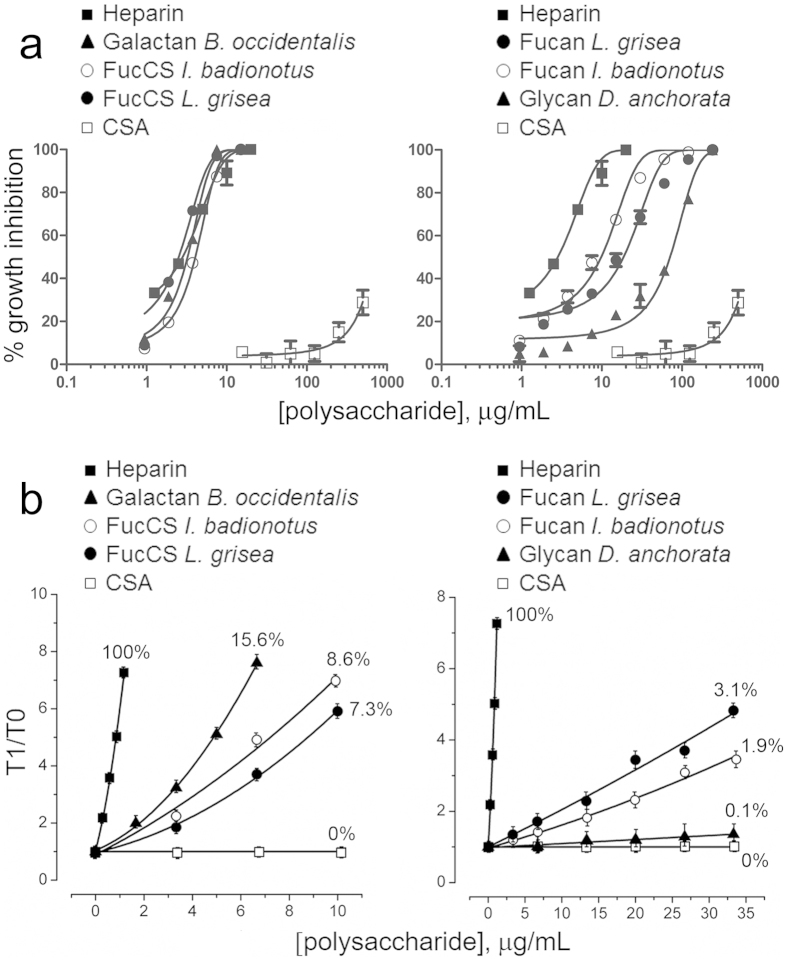
*In vitro* analysis of antimalarial and anticoagulating activities of sulfated polysaccharides. **(a)** Growth inhibition assays of *P. falciparum* cultures. **(b)** Activated partial thromboplastin time assay of anticoagulant activities, expressed as the ratio between clotting times in the presence (T1) and absence (T0) of polysaccharides. Percentages represent the respective anticlotting activities relative to that of heparin. CSA is used as a negative control for its lack of anticoagulating activity. The results are shown as the means of three independent experiments; the error bars represent standard deviations.

The antimalarial activity of the polysaccharides was analyzed in *in vitro* cultures of *P. falciparum* (Fig. 3a), revealing for most of them a significant inhibition of the parasite’s growth, with IC50s between 2.3 and 20.3 μg/mL (Table 1). These activities were similar to those found for different heparin batches (between 4 and 18 μg/mL according to our own data)^39^. The sole exception was the *D. anchorata* glycan, whose antimalarial activity was found to be relatively low, with an IC50 ~66 μg/mL. No correlation was found between *Plasmodium* growth inhibition and polysaccharide size, since the two best activities were for the largest and smallest structures corresponding, respectively, to the *B. occidentalis* galactan (~700 kDa) and the *L. grisea* FucCS (~30 kDa). The sulfated polysaccharides from marine organisms assessed here, especially the fucosylated chondroitin sulfates and sulfated fucans from sea cucumbers, have been showing remarkably homogenous structures, as demonstrated by their coincident NMR spectra obtained from different preparations^29^,^35^,^40^. Therefore, if these polysaccharides are extracted and purified properly, no significant variations in structure, and thus in pharmacological properties, are expected between batches.

According to the activated partial thromboplastin time (APTT) determined *in vitro* for the sulfated polysaccharides used in this work (Fig. 3b and Supplementary Table 1), the three best antimalarial compounds (both FucCSs and the galactan) were the most anticoagulant and the three polymers more innocuous for *Plasmodium* (both fucans and the sponge glycan) exhibited the worst anticlotting activities. Nevertheless, all compounds had comparatively small anticoagulant activities never above 16% of that from heparin, as demonstrated by their significantly higher doses necessary to double the control APTT (Supplementary Table 1), which suggests that anticoagulating and antimalarial activities are not directly related.

### Sulfated polysaccharides inhibit *Plasmodium* invasion of red blood cells

**Figure 4:**
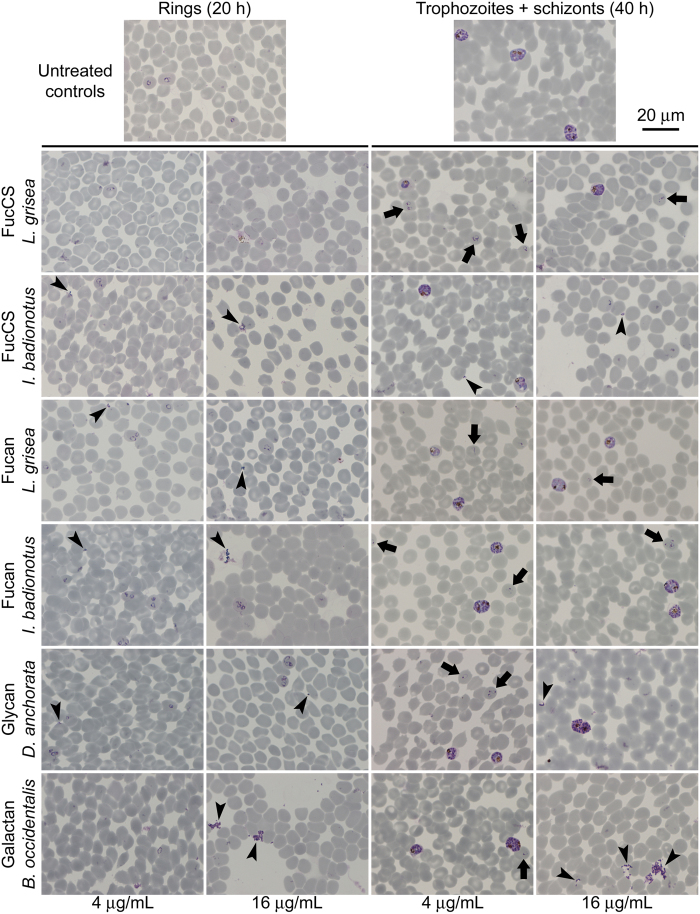
Microscopic images of Giemsa-stained *in vitro* pRBC cultures of *P. falciparum* treated with sulfated polysaccharides in an invasion inhibition assay. Pictures corresponding to the cycle phases when rings and trophozoites+schizonts are the dominant forms expected were taken, respectively, 20 and 40 h after treatment. Arrows and arrowheads indicate examples, respectively, of ring stages and of merozoites that have failed to invade pRBCs.

**Figure 5:**
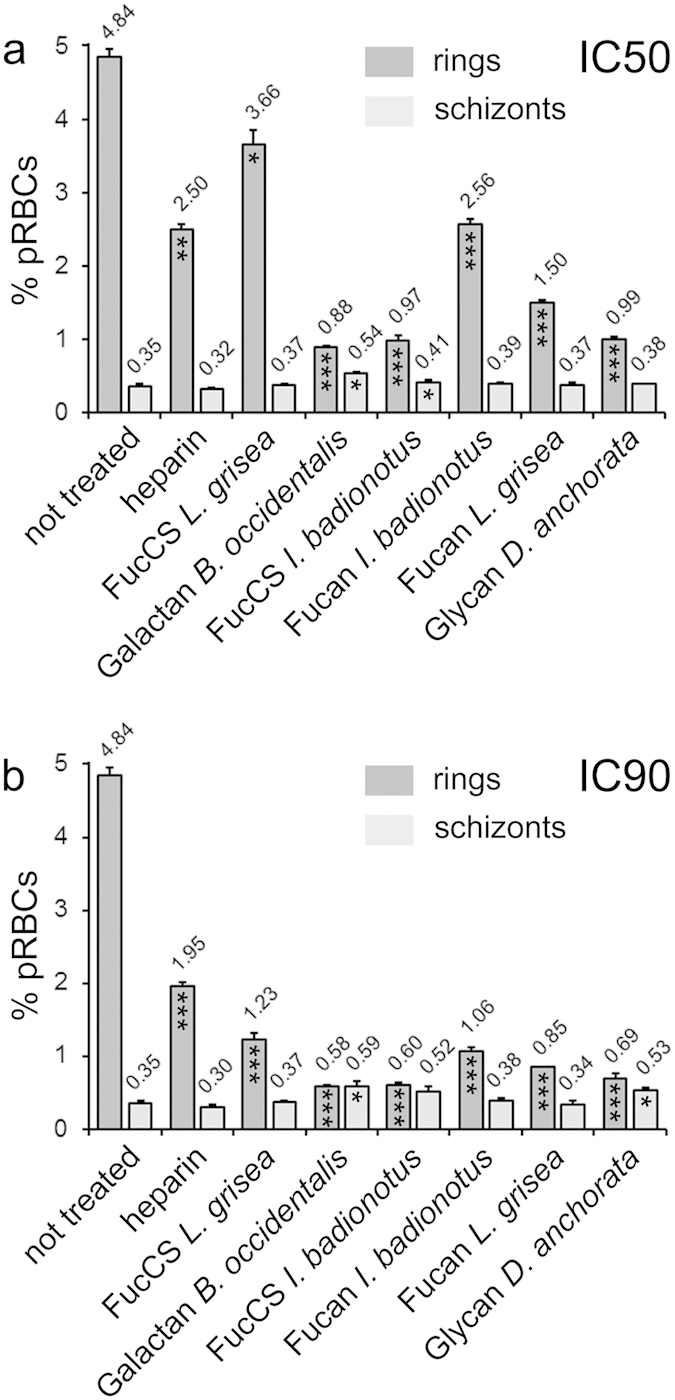
Flow cytometry analysis of the inhibition of red blood cell invasion by *P. falciparum* in the presence of sulfated polysaccharides. The values indicate the corresponding absolute percentages (parasitemias) of rings and schizonts in the cultures, 20 h after treatment and at polysaccharide concentrations corresponding to the respective (a) IC50 and (b) IC90 reported in Table 1. The results are shown as the means of three independent experiments; the error bars represent standard deviations. Significant differences in the numbers of rings and schizonts relative to the respective non-treated controls as determined by *t*-tests are indicated by asterisks (*p<0.05, **p<0.005, ***p<0.001).

Since the antimalarial mechanism of heparin and related polysaccharides had been described to operate through inhibition of the invasion of RBCs by *Plasmodium*, we proceeded to investigate the invasion inhibition activity of the marine sulfated polysaccharides. Late-stage pRBC cultures that had been treated with the different structures revealed upon microscopic examination at 20 h post-treatment a clear decrease in ring stages relative to untreated samples (Fig. 4). Polysaccharide-treated samples thus showed a delay in *P. falciparum* development, as evidenced by the presence at 40 h within the intraerythrocytic cycle of a significant fraction of ring stages relative to untreated controls which contained, as expected, trophozoites and schizonts only. Quantitative microscopic counts evidenced a clear decrease in the invasion rate of all polysaccharide-treated samples (Table 2). Maturation rates, on the other hand, were not negatively affected, indicating that if rings are formed, their progression towards trophozoites and schizonts seems to proceed normally. The observation that some of the samples had maturation rates larger than the untreated control suggests that these cases reflected the presence of a significant number of parasites that either completed their invasion or started their differentiation into identifiable rings after the count of ring stages was made. A clear example of this is represented by *L. grisea* FucCS-treated samples, whose parasitemias at 40 h post-treatment were higher than those expected from the low invasion rate reported for this polysaccharide in Table 2. Likely, the reduced ring numbers observed in microscopic counts of *in vitro* assays indicates a slower invasion process of otherwise viable merozoites. These retarded invasions (therefore not detected as ring stages) eventually develop into trophozoites, which results in an apparently high maturation rate from rings to late forms. Indeed, when multiplying invasion by maturation rates, which gives an approximate comparative estimation of parasite viability, the values obtained (not shown) are in good agreement with the respective inhibitory effects on parasite growth (Fig. 3), with *L. grisea* FucCS exhibiting the highest antiplasmodial activity when all polysaccharides are tested at 4 μg/mL. Flow cytometry analyses of *P. falciparum* cultures treated at late stage with sulfated polysaccharides confirmed a clear dose-dependent invasion inhibition at their respective IC50 (Fig. 5a) and IC90 (Fig. 5b).

The accumulated experimental evidence indicates that invasion inhibition is the antimalarial mechanism through which sulfated polysaccharides operate. However, the short time that free merozoites are present in the blood circulation suggests that the process of parasite binding might occur, at least in part, inside pRBCs. Following previously established protocols^39^, we added fluorescein-labeled heparin to live pRBC cultures and after 30 min of incubation the samples were processed for confocal fluorescence microscopy analysis. The resulting data show that heparin added to living pRBC cultures not only specifically targeted pRBCs vs. RBCs *in vitro*, but it entered live pRBCs and bound intraerythrocytic developing merozoites (Supplementary Fig. 1).

### *In vivo* antimalarial activity analysis of sulfated polysaccharides

**Figure 6:**
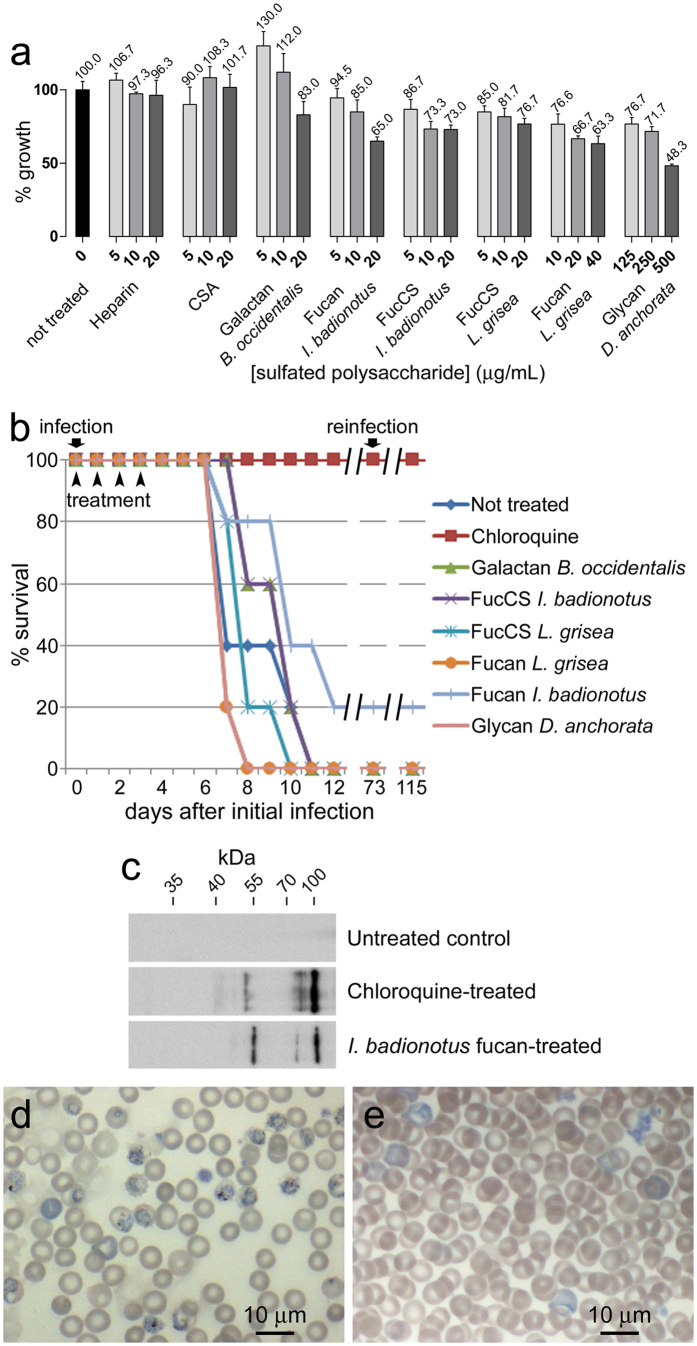
Cytotoxicity and *in vivo* antimalarial activity analyses of sulfated polysaccharides. (**a**) Unspecific toxicity for human umbilical vein endothelial cell cultures of sulfated polysaccharides assayed at concentrations around their respective IC50 for *P. falciparum* growth *in vitro*. The results are shown as the means of three independent experiments; the error bars represent standard deviations. (**b**) Kaplan-Meier plot for the *in vivo* assay of the effect on *P. yoelii*-infected mice (n = 5 animals/sample) of polysaccharides administered iv at the estimated μg mL^−1^ day^−1^ indicated in Table 3. Chloroquine was administered iv as a positive control at a dose of 5 mg kg^−1^ day^−1^. (**c**) Western blot for the detection of IgGs against *P. yoelii* antigens in the serum of surviving infected mice that had been treated 35 days before with chloroquine or with the *I. badionotus* fucan. The untreated control corresponds to a noninfected mouse of the same age. (**d,e**) Microscope images of blood samples from the surviving *I. badionotus* fucan-treated mouse at (**d**) day 4 and (**e**) day 40 after reinfection without further treatment.

*P. yoelii*-infected mice were treated iv with polysaccharide doses selected after consideration of their *in vitro* antimalarial activity, anticoagulation capacity, and unspecific cytotoxicity. Although a significant toxicity in endothelial cell cultures was found at some of the administered doses for most marine sulfated polysaccharides when compared to the same heparin concentrations (Fig. 6a and Supplementary Table 2), no adverse effects were observed in the animals during the first week of the assay apart from the symptoms characteristic of a malaria infection. Except for *D. anchorata* glycan and *L. grisea* fucan, all compounds reduced parasitemia when compared to untreated controls (Table 3). *I. badionotus* fucan provided the best improvement in mice survival (Fig. 6b), and in one animal treated with this compound parasitemia was reduced from 10.4% at day 4 to undetectable levels. Western blot analysis revealed the presence of antibodies against *P. yoelii* antigens in the plasma of surviving mice (Fig. 6c). To explore if the observed increased antibody titers were consequence of an immune response against the parasite, the surviving animals were re-inoculated with *P. yoelii* 73 days after the initial infection; all mice, including that treated with the *I. badionotus* fucan, survived the new infection without any treatment (Fig. 6b). Microscopic observation of blood smears confirmed the infection of the latter animal, which at day 4 had 18.3% parasitemia (Fig. 6d), and the progressive reduction of parasitemia until its complete elimination (Fig. 6e). All surviving animals were alive and without symptoms of disease at day 42 after the second, untreated infection.

## Discussion

Previous work had demonstrated that the presence of sulfate groups was paramount for the binding of *L. grisea* FucCS to human lung endothelial cells and placenta cryosections under static and flow conditions^14^, and that sulfated FucCS was capable of inhibiting pRBC cytoadherence in these cell models. Because pRBC sequestration in the microvasculature of vital organs plays a key role in the pathogenesis of cerebral and pregnancy malaria, *L. grisea* FucCS has been proposed for the treatment of severe disease. The crucial role of sulfate groups in the context of malaria was further evidenced by the ability of *L. grisea* FucCS to disrupt *P. falciparum* rosettes, which was significantly lost upon desulfation^14^. Other evidences illustrating the physiological importance of sulfate groups came from reports showing that their removal abolished the antithrombotic and anticoagulant effects of FucCS^33^, and that their presence was essential for preserving the inhibitory effects of the polysaccharide in interactions mediated by P- and L-selectin^41^. The *in vitro* data reported here show that polysaccharides containing α-fucose as internal units are less active as antimalarials than polymers having α-fucose as branches. The sulfated fucan from *I. badionotus* was found to have a slightly higher *in vitro* antimalarial activity than that of *L. grisea*, probably because of the additional 2-sulfation. The observation that both FucCSs have similar *in vitro* antimalarial effect despite the marked differences in their α-fucose-containing branches suggests that, beyond a minimal threshold, the presence of additional 2,4-disulfated fucose units does not result in higher antiplasmodial potency, as it was similarly reported for the anticoagulant activity of FucCS^35^.

Compared to heparin, marine sulfated polysaccharides exert their antimalarial activity *in vitro* at concentrations where their anticoagulant activity is low. The semi-synthetic heparin-like polysaccharide K5-NSOS-H also showed high antiplasmodial activity despite of its low anticoagulant capacity^16^, related to its lack of iduronic acid units which are essential to promote the interactions with antithrombin and heparin co-factor II^42^. However, to trace a parallel between the antimalarial and anticoagulant activities of heparin-like molecules and fucosylated chondroitin sulfates and sulfated galactan is difficult because these polysaccharides from marine organisms present serpin-independent anticoagulant properties^31^,^32^. The polysaccharides used in this work do not require fractionation and/or chemical modification after purification^31^ and, unlike heparin, are not derived from mammals, thus reducing the risk of contamination by human-affecting pathogens. These compounds are present at high concentrations in marine organisms and can be isolated with relatively high yields of at least ~1% dry weight. The synthesis of sulfated polysaccharides from the marine organisms used here is unfeasible because the enzymatic machinery for their synthesis is still unknown. However, sea cucumbers are already mass cultivated in several countries, especially in China, where they are used as food^43^. Several species of seaweeds are also commercially farmed^44^, and particularly the alga *B. occidentalis* is abundant in the northeastern coast of Brazil; almost 50% of its dry weight is sulfated galactan^45^, making its harvesting a feasible strategy.

Other already described antimalarial compounds like pentosan polysulfate, curdlan sulfate and dextran sulfate are obtained via chemical sulfation of neutral polysaccharides and show serious side effects such as thrombocytopenia, intracerebral hemorrhage and colitis^46^,^47^. Curdlan sulfate, which has been proposed as adjunct medication to conventional therapy in patients with severe malaria, has been described to possess as adverse effect an increase in APTT^48^. Fucoidan, also reported to have an inhibitory effect on *Plasmodium* growth^49^, had a certain level of toxicity for a murine macrophage cell line and was described to occasionally cause eye hemorrhages and death of the animals^49^. Although at concentrations close to their *in vitro* IC50s marine sulfated polysaccharides exhibited significant toxicity in endothelial cell cultures, higher *in vivo* amounts did not trigger observable adverse effects in mice during the first week of treatment. Consistently, FucCS can be satisfactorily administered orally^50^, without toxic or cumulative effects in tissue observed after daily doses to animals of 50 mg/kg for 30 days (Mourão, unpublished data). Nevertheless, the potential unspecific toxicity of sulfated polysaccharides in future antimalarial clinical applications can possibly be averted by encapsulating them in pRBC-targeted nanocapsules as it has been reported for other antimalarial agents^51^,^52^,^53^. In the case of heparin, the polysaccharide itself has been demonstrated to be capable of acting as targeting molecule of drug-loaded nanocarriers^39^, thus adding to its own antiparasitic action and potentiating therapeutic activity. Some of the pernicious effects of sulfated polysaccharides, such as the anticoagulant activity of heparin, are significantly reduced when immobilized on a substrate^54^. Surface plasmon resonance biosensor studies showed that covalent binding through its carboxyl groups dramatically reduced the interaction of heparin with antithrombin III^55^. Conjugation to nanoparticles can thus be explored as an interesting approach to reduce potential toxic side-effects.

We have observed that formation of ring-stage parasites is clearly reduced in the presence of sulfated polysaccharides, in agreement with preexisting data indicating that their antimalarial activity unfolds by inhibition of merozoite invasion^8^,^9^,^13^,^14^,^15^,^16^,^49^,^56^,^57^,^58^. The mechanism through which this invasion blocking proceeds has not been elucidated yet, although the finding that sulfation patterns are crucial for the inhibitory effect of heparin and similar compounds^16^, suggests that it is the result not only of nonspecific ionic interactions but also of particular conformations of anions present in the polysaccharides^8^. Whereas binding of heparin to merozoites has been described to be mediated by multiple protein receptors^17^,^18^, GAG-pRBC associations are mainly based on interactions with the parasite-derived adhesin, *P. falciparum* erythrocyte membrane protein 1, PfEMP1^59^. The subsequent internalization of heparin into pRBCs might be an unspecific uptake through the tubulovesicular network induced by *Plasmodium* during its intraerythrocytic growth^60^. Such entry into pRBCs and coating of developing merozoites before they egress, permits the invasion inhibition activity of heparin to be manifested since the first moment when *Plasmodium* cells are free in the blood circulation. This is important regarding possible future therapeutic applications of sulfated polysaccharides; if the observed activity were only exerted upon binding to free, extraerythrocytic merozoites, their rapid invasion of RBCs^61^ would severely compromise clinical applications. Because heparin is capable of penetrating live pRBCs and of binding intracellular merozoites^39^, heparin-based antimalarial therapies can be administered during the wide time frame when late stages are present in clinical malaria. Smaller fragments of marine polysaccharides might also have this behavior, although the finding that the ~700 kDa *B. occidentalis* galactan has antimalarial activity similar to that of the ~30–40 kDa FucCSs from *L. grisea* and *I. badionotus* suggests that pRBC internalization of the polymers might not be essential for their capacity to inhibit *Plasmodium* growth.

Experimental evidence presented here and elsewhere^7^,^8^,^9^,^10^,^11^,^12^,^13^,^14^,^15^,^16^ has shown that pRBCs and merozoites are targeted by different sulfated polysaccharides, and that heparin targets pRBCs and merozoites from widely diverging malarias (e.g. human-infecting *P. falciparum* and the murine malaria parasite *P. yoelii*^39^). The widespread pathogen resistance against virtually all currently used drugs^6^ and the difficulties in selecting heparin-resistant parasites^16^, places antimalarial sulfated polysaccharides as interesting candidate molecules deserving careful exploration.

## Methods

### Ethics statement

All experiments were performed in accordance with the corresponding relevant guidelines and regulations. The studies reported here were performed under protocols reviewed and approved by the Ethical Committee on Clinical Research from the Hospital Clínic de Barcelona (Reg. HCB/2014/0910) and the Ethical Committee on Animal Experimentation from the Barcelona Science Park (Reg. 20140917). The animal care and use protocols followed adhered to the specific national and international guidelines specified in the Spanish Royal Decree 53/2013, which is based on the European regulation 2010/63/UE. All human blood samples used for *P. falciparum in vitro* cultures were purchased from the Banc de Sang i Teixits (http://www.bancsang.net/) and irreversibly anonymized prior to their arrival.

### Materials

Except where otherwise indicated, reactions were performed at room temperature (20 °C), and reagents were purchased from Sigma-Aldrich Corporation (St. Louis, MO, USA).

### Extraction and purification of sulfated polysaccharides

Samples of the marine organisms were cut into 1 mm^3^ pieces, immersed three times in acetone and dried at 60 °C. Sulfated polysaccharides were extracted from 10 g of the desiccated tissues by extensive papain digestion, and the extracts were partially purified by cetylpyridinium and ethanol precipitations as described^62^. Approximately 100 mg dry weight of crude polysaccharide extract was obtained from each species. Extracts were applied to a high-performance liquid chromatography system-linked Mono Q column (GE Healthcare, UK), equilibrated with 5 mM ethylenediaminetetraacetic acid, 20 mM Tris-HCl, pH 7.0. The polysaccharides were eluted from the column using a 0–3 M NaCl linear gradient at a flow rate of 1 mL/min. 0.5 mL fractions were collected and checked by metachromatic assay using 1,9-dimethylmethylene blue^63^, and by measuring conductivity to estimate NaCl concentration. The fractions containing sulfated polysaccharides were pooled, dialyzed against distilled water and lyophilized, and the corresponding structures confirmed by nuclear magnetic resonance analysis as described^35^,^37^,^38^.

### *P. falciparum* cell culture and growth inhibition assays

The *P. falciparum* 3D7 strain was grown *in vitro* in group B human RBCs using previously described conditions^51^. Briefly, parasites (thawed from glycerol stocks) were cultured at 37 °C in Petri dishes containing RBCs in Roswell Park Memorial Institute (RPMI) complete medium under a gas mixture of 92% N_2_, 5% CO_2_, and 3% O_2_. Synchronized cultures were obtained by 5% sorbitol lysis, and the medium was changed every 2 days keeping 3% hematocrit. For culture maintenance, parasitemias were kept below 5% late forms by dilution with washed RBCs prepared as described elsewhere^51^. For growth inhibition assays, parasitemia was adjusted to 1.5% with more than 90% of parasites at ring stage after sorbitol synchronization. 150 μL of this *Plasmodium* culture was plated in 96-well plates and incubated in the presence of polysaccharides for 48 h in the conditions described above. Parasitemia was determined by flow cytometry, after staining pRBC DNA with the nucleic acid dye Syto 11, added 10 min before analysis. Samples were analyzed using a BD FACSCalibur™ flow cytometer and parasitemia was expressed as the number of parasitized cells per 100 erythrocytes. Acquisition was configured to stop after recording 50,000 events within the RBC population. IC50 and IC90 were derived from non-linear fit dose response curves (Log doses versus normalized inhibitions).

### Merozoite invasion inhibition assay

Synchronized cultures of *P. falciparum* 3D7 were enriched using Percoll (GE Healthcare) purification to obtain late trophozoites and early schizonts, and diluted to ~1% initial parasitemia and 3% hematocrit. Assays were performed in 24-well, flat-bottomed microculture plates where 1 mL of culture was incubated in RPMI supplemented with different amounts of each polysaccharide in study, for 20 h as described above. After incubation, smears were prepared by fixing cells in methanol for a few seconds and then staining them for 10 min with Giemsa (Merck Chemicals, Germany) diluted 1:10 in Sorenson’s Buffer, pH 7.2. Plates were incubated for another 20 h before preparing a new set of smears. Slides were observed with an optical microscope Nikon Eclipse 50i (Japan) and pictures were taken with a Nikon Digital Sight DS-U2 camera. For quantitative determinations, the cultures were analyzed by flow cytometry as described above. To assess invasion and maturation rates, respectively, the following formulae were applied:


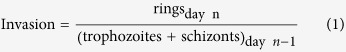



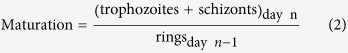


### Activated partial thromboplastin time (APTT) assay

Various concentrations of sulfated polysaccharides in 100 μL of human plasma were mixed with 100 μL of undiluted APTT reagent (kaolin bovine phospholipid reagent from Biolab-Merieux AS, Rio de Janeiro, Brazil). After incubating for 2 min at 37 °C, 100 μL of 25 mM CaCl_2_ was added to the mixture, and the clotting time was recorded in an Amelung KC4A coagulometer (Heinrich Amelung GmbH, Lemgo, Germany). The results were expressed as the clotting time ratio in the presence vs. absence of different polysaccharide concentrations. Anticoagulant activity was indicated as IU/mg using a parallel standard curve based on the 6th International Heparin Standard (2,145 units per vial, 200.04 IU/mg), obtained from the National Institute for Biological Standards and Control (Potters Bar, UK).

### Fluorescence microscopy

Living *P. falciparum* cultures with mature stages of the parasite were incubated in the presence of 10 μg/mL fluorescein-labeled heparin (Life Technologies) in phosphate buffered saline, pH 7.4 (PBS) supplemented with 0.75% bovine serum albumin, for 30 min at 37 °C with gentle stirring. After PBS washing, blood smears were prepared and cells were fixed for 20 min with 1% (v/v) paraformaldehyde in PBS. Parasite nuclei were stained with 4′6-diamino-2-phenylindole (DAPI) and the RBC membrane was labeled with wheat germ agglutinin–tetramethylrhodamine conjugate. Slides were finally mounted with ProLong® Gold antifade reagent, and analyzed with a Leica TCS SP5 laser scanning confocal microscope.

### Polyacrylamide gel electrophoresis

Electrophoresis was performed in 6% polyacrylamide gels using a Mini-Protean Tetra Cell System (Bio-Rad Laboratories Inc.). Samples containing 20 μg polysaccharide (at 1 mg/mL) were boiled in the presence of nonreducing sample buffer for 5 min, and electrophoresed at 100 V for 40 min. The gel was stained with 0.5% Alcian Blue solution in 3% acetic acid/25% isopropanol for 30 min with gentle stirring. Finally, the gel was de-stained in 10% acetic acid/40% ethanol overnight and digitalized.

### Unspecific cytotoxicity assay

Human umbilical vein endothelial cells (primary culture provided by Dr. Francisco J. Muñoz, Pompeu Fabra University, Barcelona, Spain) were seeded in 96-well plates at a density of 5000 cells/100 μL/well, and incubated for 24 h at 37 °C in Medium 199 with Earle’s salts, supplemented with L-glutamine and fetal calf serum (FCS; LabClinics). After that time the medium was carefully removed with a pipette, and 100 μL of new medium (without FCS in this case) containing different sample concentrations was added. After incubating for 48 h at 37 °C, 10 μL of the cell proliferation assay reagent WST-1 (Roche Life Science) was added, and 1 h later absorbance was measured at 440 nm.

### Antimalarial activity assay *in vivo*

The *in vivo* antimalarial activity of sulfated polysaccharides was studied in a 4-day blood suppressive test as previously described^64^. Briefly, Balb/C female mice (n = 5/sample; Janvier Laboratories) were inoculated intraperitoneally with 2 × 10^6^ RBCs extracted from an animal infected by the *Plasmodium yoelii yoelii* 17 XL lethal strain. Treatment with the antimalarial drug chloroquine (5 mg kg^−1^ day^−1^)^65^ or polysaccharides dissolved in PBS started 2 h later (day 0) with a 200-μL single dose administered intravenously, followed by identical dose administration for the next 3 days. To obtain the desired final *in vivo* doses, the compounds were prepared 10× concentrated, assuming a volume of 2 mL of circulating mouse blood. A control untreated group received PBS. Activity was determined by microscopic counting at day 4 of blood smears stained with Wright’s solution (Merck Chemicals). Mice were fed a commercial diet *ad libitum* and treated with humane care, being euthanized if reaching a 20% weight loss for two consecutive days. The sacrifice method was exposure to 95% CO_2_ following anesthesia with 5% isoflurane vaporized in O_2_. On day 73 after the initial inoculation those surviving animals were re-inoculated as described above. The mice did not receive any treatment after this second infection and parasitemia was monitored by blood smear counting.

### *P. yoelii* protein extraction from infected whole blood

Protein lysates were extracted from the whole blood of infected Balb/C female mice having >50% parasitemia. Blood was collected in Microvette® CB 300 tubes (Sarstedt, Germany) and kept at −80 °C until protein extraction. RBC lysis was performed by adding 10× vol of saponin 0.1% (w/v) in PBS. After washing twice with cold PBS, the pellet was treated with 2 vol of extraction buffer consisting of 50 mM NaCl, 0.5% Mega 10, 3% CHAPS, and 50 mM Tris-HCl, pH 8.0, supplemented with a protease inhibitor cocktail (Roche). The samples were subjected to four freeze-thaw cycles, and the lysates were finally centrifuged at 20,000 g for 30 min (4 °C). Protein concentration was determined by the DC protein assay (Bio-Rad), and *P. yoelii* total protein samples were stored at −20 °C until use.

### Western blot

10 μg of *P. yoelii* total protein extract were fractionated in a reducing 10% SDS-PAGE (Bio-Rad), transferred to polyvinylidene difluoride membranes and blocked with Z buffer (100 mM MgCl_2_, 0.5% Tween 20, 1% Triton X-100, 1% BSA,100 mM Tris-HCl, pH 7.4, supplemented with 5% FCS). Membranes were then incubated at 4 °C overnight with 1:10,000 dilutions of sera from the mice that survived the infection, followed by a 1-h incubation with secondary horseradish peroxidase-labeled anti-mouse IgG (Amersham Biosciences) at a 1:10,000 dilution.

### Statistical analysis

Data are presented as the mean ± standard deviation of at least three independent experiments, and the corresponding standard deviations in histograms are represented by error bars. Cell counts from Giemsa-stained slides were done using the Plasmoscore programme. The parametric Student’s t-test was used to compare two independent groups when data followed a Gaussian distribution, and differences were considered significant when *p* ≤0.05. Percentages of viability were obtained using non-treated cells as control of survival and IC_50_ values were calculated by nonlinear regression with an inhibitory dose-response model using GraphPad Prism5 software (95% confidence interval). Concentrations were transformed using natural log for linear regression. Regression models were adjusted for replicates and assay data. In anticoagulant assays, the polysaccharide concentrations (mean ± standard deviation) necessary to double the control (T0) APTT^66^ were compared via one-way ANOVA with Tukey´s test using the software Origin-Pro 8.0 (OriginLab).

## Additional Information

**How to cite this article**: Marques, J. *et al.* Marine organism sulfated polysaccharides exhibiting significant antimalarial activity and inhibition of red blood cell invasion by *Plasmodium.* *Sci. Rep.* **6**, 24368; doi: 10.1038/srep24368 (2016).

## Supplementary Material

Supplementary Information

## Figures and Tables

**Table 1:** IC50 and IC90 values derived from growth inhibition assays of *P. falciparum* cultures.

Polysaccharide	IC50 (μg/mL)	IC90 (μg/mL)
Heparin	4.1	8.0
FucCS *L. grisea*	2.3	5.5
Galactan *B. occidentalis*	3.5	6.8
FucCS *I. badionotus*	4.2	7.7
Fucan *I. badionotus*	9.5	24.8
Fucan *L. grisea*	20.3	48.8
Glycan *D. anchorata*	66.3	152.0
CSA	>1000	>1000

**Table 2:** Invasion and maturation rates corresponding to the invasion inhibition assay from Fig. 4.

Compound	Invasion rate	Maturation rate
Not treated	3.60 ± 0.10	0.96 ± 0.06
FucCS *L. grisea*	0.32 ± 0.02***	2.73 ± 0.09**
Galactan *B. occidentalis*	1.10 ± 0.10***	1.14 ± 0.08
FucCS *I. badionotus*	1.00 ± 0.19**	1.50 ± 0.20
Fucan *I. badionotus*	2.61 ± 0.04**	1.05 ± 0.05
Fucan *L. grisea*	1.74 ± 0.38*	1.75 ± 0.09**
Glycan *D. anchorata*	1.93 ± 0.55*	1.19 ± 0.34

The data are derived from microscopic counting at, respectively, 20 and 40 h post-treatment of the samples incubated with 4 μg/mL of sulfated polysaccharides. The results are shown as the means of three independent experiments ± standard deviation, with 900 cells counted for each sample (300 × 3 replicates). Significant differences relative to non-treated control as determined by *t*-tests are indicated by asterisks (*p<0.05, **p<0.005, ***p<0.001).

**Table 3:** Estimated compound concentrations in blood and determined day 4 parasitemias for the *in vivo* assay from Fig. 6b.

Treatment	μg compound mL^−1^ day^−1^	Day 4 parasitemia
Not treated	–	19.9 ± 9.3
Chloroquine	5.0	1.4 ± 0.2
Galactan *B. occidentalis*	5.6	18.4 ± 1.3
FucCS *I. badionotus*	8.0	14.0 ± 1.1
FucCS *L. grisea*	10.0	11.0 ± 5.2
Fucan *L. grisea*	11.0	22.3 ± 5.5
Fucan *I. badionotus*	15.0	14.3 ± 5.6
Glycan *D. anchorata*	125.0	21.6 ± 2.8

These daily administered doses assume 2 mL of blood in a mouse into which were injected 200 μL of 10× concentrated compound solutions. The results are shown as the means of five independent experiments ± standard deviation.
