# Supplementary material for: Marine organism sulfated polysaccharides exhibiting significant antimalarial activity and inhibition of red blood cell invasion by Plasmodium
Source: Sci Rep. 2016 Apr 13;6:24368. doi: 10.1038/srep24368 (PMC4829872; doi:10.1038/srep24368)
Supplement: Supplementary Information [file srep24368-s1.doc]

**Marine organism sulfated polysaccharides exhibiting significant antimalarial activity and inhibition of red blood cell invasion by *Plasmodium***

Joana Marques^1-3^, Eduardo Vilanova^4^, Paulo A. S. Mourão^4^ and Xavier Fernàndez-Busquets^1-3^*

^1^Nanomalaria Group, Institute for Bioengineering of Catalonia (IBEC), Barcelona, Spain. ^2^Barcelona Institute for Global Health (ISGlobal), Barcelona Center for International Health Research (CRESIB, Hospital Clínic-Universitat de Barcelona), Barcelona, Spain. ^3^Nanoscience and Nanotechnology Institute (IN2UB), University of Barcelona, Spain. ^4^Hospital Universitário Clementino Fraga Filho and Instituto de Bioquímica Médica, Universidade Federal do Rio de Janeiro, Brazil.

* Corresponding author

E-mail: [xfernandez_busquets@ub.edu](mailto:xfernandez_busquets@ub.edu)


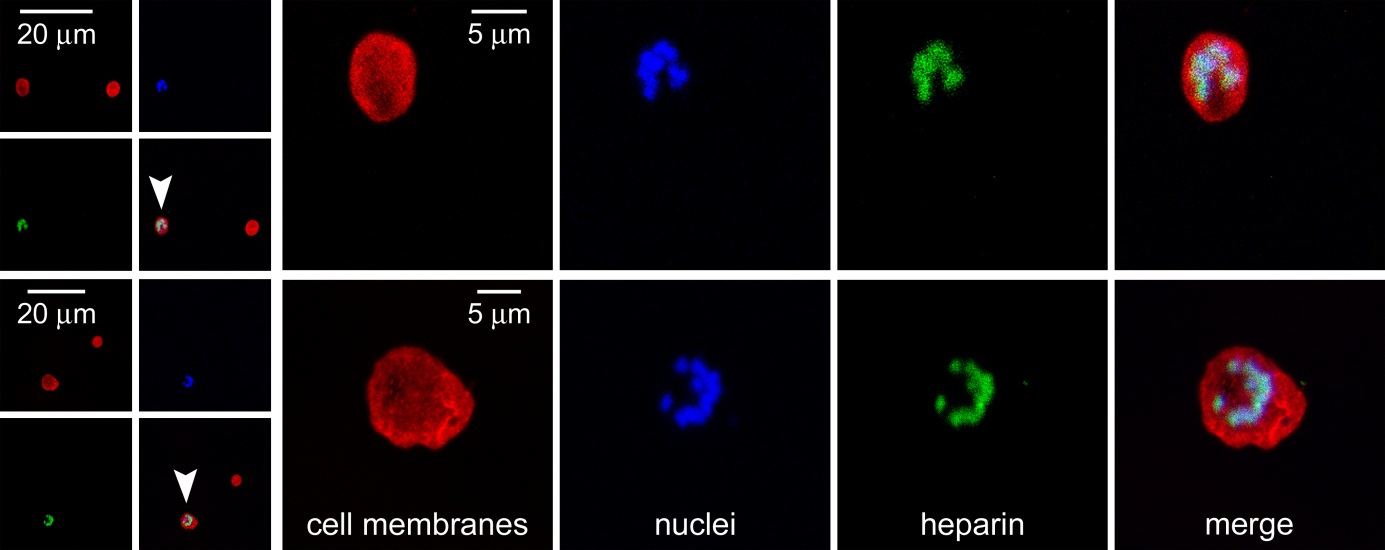


**Supplementary Figure 1. Binding of heparin to *P. falciparum* merozoites inside pRBCs.** Fluorescein-labeled heparin was added to pRBC cultures that were processed for fluorescence microscopy after 30 min of incubation. The four small panels to the left of each series are included to show pRBC vs. RBC specificity. Arrowheads indicate pRBCs (revealed by DAPI stain of *Plasmodium* DNA within the otherwise anucleated RBC) imaged at higher resolution in the right-hand panels.

| **Polysaccharide** | **Concentration required to double the APTT (µg/mL)** |
| --- | --- |
| Heparin | 0.261 ± 0.002 |
| FucCS *L. grisea* | 3.562 ± 0.021 |
| Galactan *B. occidentalis* | 1.671 ± 0.008 |
| FucCS *I. badionotus* | 3.023 ± 0.015 |
| Fucan *I. badionotus* | 13.40 ± 0.052 |
| Fucan *L. grisea* | 8.433 ± 0.052 |
| Glycan *D. anchorata* | 55.50 ± 0.422 |
| CSA | >100 |

**Supplementary Table 1. Anticoagulant potencies of the sulfated polysaccharides.** The activated partial thromboplastin time (APTT) values are shown as the means of three independent experiments ± standard deviations. All anticoagulant activities were significantly lower than that of heparin (*p<*0.001 by one-way ANOVA using Tukey´s test).

| **Polysaccharide** | **5 µg/mL** | **10 µg/mL** | **20 µg/mL** |
| --- | --- | --- | --- |
| FucCS *L. grisea* | 0.00054 | 0.024 | 0.00029 |
| Galactan *B. occidentalis* | 0.22 | 0.036 | 0.00026 |
| FucCS *I. badionotus* | 0.0022 | 0.033 | 0.00042 |
| Fucan *I. badionotus* | 0.31 | 0.02 | 0.00037 |
| Fucan *L. grisea* | - | 0.04 | 0.001 |
| Glycan *D. anchorata* | - | - | - |

**Supplementary Table 2. Significance of differences, relative to the same concentrations of heparin, in the cytotoxicity of marine sulfated polysaccharides reported in Fig. 6a.** The table shows the corresponding p values as determined by *t*-tests.
